# Sagittal abdominal diameter shows better correlation with cardiovascular risk factors than waist circumference and BMI

**DOI:** 10.1186/2251-6581-12-41

**Published:** 2013-07-15

**Authors:** Natalia Cavalheri de Souza, Erick Prado de Oliveira

**Affiliations:** 1 Paulista University (UNIP), Bauru, São Paulo, Brazil; 2 School of Medicine, Federal University of Uberlândia, Uberlândia, Minas Gerais Brazil

**Keywords:** Waist circumference, Sagittal abdominal diameter, Blood pressure, Glycemia, Dyslipidemia

## Abstract

**Background:**

Obesity (abdominal adiposity) is a risk factor for cardiovascular diseases and the most used methods to measure the adiposity are body mass index (BMI), waist circumference (WC), and sagittal abdominal diameter (SAD).

**Objective:**

To correlate BMI, WC, and SAD with biochemical parameters and blood pressure in adults.

**Methods:**

A non-experimental exploratory/descriptive and cross sectional study was developed and it was assessed 133 subjects (59 men and 74 women) aging between 18 and 87 years. It was registered the patients’ weight (kg), height (m), BMI (kg/m^2^), WC (cm) and SAD (cm), and these parameters were correlated with glycemia, triglycerides, total cholesterol, HDL-c, LDL-c and blood pressure.

**Results:**

After adjustment for gender and age, it was observed a positive correlation between SAD and systolic arterial blood pressure (r = 0.20), glycemia (r = 0.20), triglycerides (r = 0.32), LDL (r = 0.26), total cholesterol (TC) (r = 0.33), and a negative correlation with HDL-c (r = −0.21) (p < 0.05). It was observed a positive correlation between WC and systolic arterial blood pressure (r = 0.14), triglycerides (r = 0.31), total cholesterol (r = 0.21), and a negative correlation with HDL-c (r = −0.24) (p < 0.05). BMI showed a positive correlation with systolic arterial blood pressure (r = 0.22), total cholesterol (r = 0.20), and triglycerides (r = 0.23) (p < 0.05).

**Conclusion:**

SAD correlated with almost all the cardiovascular risk factors analyzed and it might be considered the best predictor of abdominal fat and cardiovascular risk.

**Electronic supplementary material:**

The online version of this article (doi:10.1186/2251-6581-12-41) contains supplementary material, which is available to authorized users.

## Background

According to the World Health Organization, obesity is characterized by the accumulation of body fat and might be responsible for the appearance of non-transmissible chronic diseases (NTCD) [1]. According to data gathered in 2001, about 60% of deaths were caused by NTCD [2].

In general, body mass index (BMI) is used to verify if subjects are overweight or obese [3], without considering muscle mass. It was observed that high BMI and advanced age increased the predisposition to hypertension and diabetes mellitus (DM), which are two metabolic syndrome (MS) components that are also associated to cardiovascular disease (CVD) [4].

Abdominal adiposity is also a risk factor for the development of CVDs [5, 6], glucose intolerance [7, 8], hypertension and dyslipidemia [9]. Hence, it is necessary to measure and quantify abdominal adiposity [10].

Computed tomography, magnetic resonance and dual-energy x-ray absorptiometry are considered “gold standard” to assess abdominal adiposity. However, these methods are expensive and also expose subjects to radiation [11, 12].

Therefore, some anthropometric measuring methods like waist circumference (WC) and sagittal abdominal diameter (SAD) [3] are used as abdominal obesity markers due to the fact that they offer lower costs, and are not only easy to measure but also harmless. They are also more applicable in clinical practice as well as in epidemiologic studies [13, 14].

Waist circumference is the measurement that is more commonly used to verify abdominal adiposity [15] because it is easy to be assessed by any person that received little training and may be used to predict MS [16, 17]. However, WC presents some limitations, because there are subjects whose abdominal adiposity seems to move the umbilical cord characterizing an “apron type belly” [18].

SAD seems to be the best predictor of abdominal adiposity regarding cardiovascular morbity and mortality [19]. When SAD is measured with the subject at supine position, fat slides to the sides of the waist, thus reflecting visceral adipose tissue [20], and making it a more efficient method than the WC [16, 21, 22]. WC as well as SAD, has specific measurement sites: the narrowest waist measurement between thorax and waist [23], the highest abdominal diameter site [24], umbilical level [25] and the midpoint of iliac crests [26]. The last one is the most commonly used [26, 27] and it coincides with the location of L4 and L5 [28, 29].

Hence, this study aims to correlate SAD, WC, and BMI with biochemical parameters as well as arterial blood pressure of adults.

## Methods

A non-experimental exploratory/descriptive and cross sectional study was developed and it assessed 133 subjects (59 men and 74 women) aging between 18 and 87 years in the cardiology and general clinic ambulatory at “Dr. Jacob Casseb” health care Center in Agudos, São Paulo, Brazil. All the subjects signed a free-consent form, and the research project was approved by the Research Ethics Committee (document no. CEP 191.038) of the Paulista University (UNIP), Brazil.

### Anthropometry

Body weight was measured with barefooted individuals wearing as few clothes as possible. As for height, it was measured with barefooted subjects with their feet together and heads held up high [30]. Both were measured using a digital scale with a *Welmy* built-in stadiometer.

BMI was calculated dividing their weight by their height and it was classified according to World Health Organization, 2000 [31].

WC was measured using a Sanny inelastic and flexible tape measure with the individual at a standing position. It was assessed at the midpoint between the last rib and iliac crest [31].

SAD was measured using an abdominal caliper (Holtain Kahn Abdominal Caliper) with the individual lying down in supine position at the iliac crests’ midpoint. One caliper arm was placed at the back of the subject and the other one was placed on his/her abdomen [26]. The adopted cutoffs to indicate cardiovascular risk were > 23.1 cm for men and > 20.1 cm for women [32].

### Biochemical data and arterial blood pressure (ABP)

Biochemical data and ABP values were obtained from the patients’ records, and it was taken into consideration only the examinations that were performed up to one month prior to the experiment’s data collection. Enzyme colorimetric assay kits were used to quantify total cholesterol, glycemia, triglycerides (TG) and high density lipoprotein (HDL-c). Low density lipoprotein (LDL-c) was calculated by Friedewald equation [33].

ABP was assessed according to the VI Brazilian Arterial Hypertension guidelines (2010) [34].

### Statistical analysis

Data were expressed in mean ± standard deviation. Sample normality was tested by the Shapiro-Wilk test. It was used the Student’s t test to compare the characteristics of individuals of both genders. Pearson correlation (with adjustment for gender and age) was used to correlate BMI, WC, and SAD with biochemical and arterial blood pressure parameters. Significance level was set at p < 0.05 and the software STATISTICA 6.0 was used for statistical analysis.

## Results

The present study assessed adult men and women. Men showed higher values for weight and height when compared to women and their BMI was classified as overweight. WC and SAD were altered; and Systolic arterial blood pressure (SAP) as well as Diastolic arterial blood pressure (DAP) were appropriate. Glycemia, LDL-c, total cholesterol and triglycerides were altered and HDL was adequate. Women showed a higher HDL-c concentrations than men (Table 1).

**Table 1:** **Characteristics of the individuals**

Variables	Total	Men	Women	p value
Age (years)	56.9 ± 15.4	58.2 ± 15.4	56.0 ± 15.4	0.41
Weight (kg)	76.3 ± 15.2	82.4 ± 15.4	71.4 ± 13.1	0.00
Height (m)	1.6 ± 0.1	1.7 ± 0.1	1.6 ± 0.1	0.00
BMI (kg/m^2^)	29.0 ± 5.1	28.3 ± 4.7	29.5 ± 5.3	0.18
WC (cm)	101.2 ± 11.2	102.5 ± 10.1	100.2 ± 11.9	0.25
SAD (cm)	24.8 ± 4.3	24.9 ± 4.0	24.8 ± 4.6	0.84
SAP(mmHg)	128.0 ± 16.9	129.8 ± 15.6	126.6 ± 17.8	0.28
DAP (mmHg)	81.9 ± 12.7	82.2 ± 11.3	81.6 ± 13.8	0.79
Glycemia (mg/dl)	118.1 ± 54.4	116.7 ± 46.7	119.3 ± 60.1	0.78
HDL-c (mg/dl)	45.6 ± 8.7	43.5 ± 8.6	47.3 ± 8.5	0.02
LDL-c (mg/dl)	132.1 ± 31.8	1276 ± 31.0	135.4 ± 32.3	0.22
Total cholesterol (mg/dl)	206.5 ± 37.2	202.8 ± 36.5	209.5 ± 37.8	0.33
Triglycerides (mg/dl)	168.2 ± 114.8	187.9 ± 142.1	152.1 ± 84.1	0.09

Abbreviations: *WC* Waist circumference, *SAD* Sagittal abdominal diameter, *SAP* Systolic arterial blood pressure, *DAP* Diastolic Arterial blood pressure, *HDL* High density lipoprotein, *LDL* Low density lipoprotein, *BMI* body mass index.

**Figure 1:**
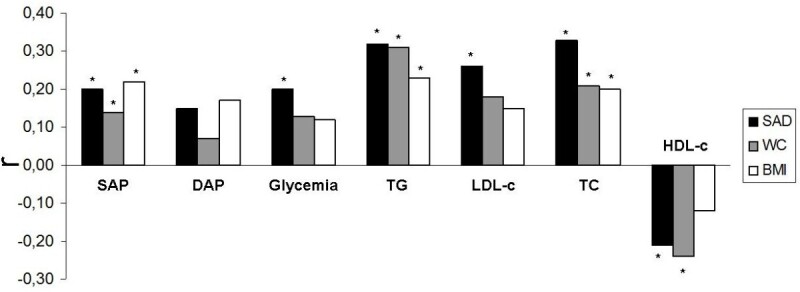
**Biochemical variables and arterial blood pressure correlations with SAD, WC, and BMI (adjusted for gender and age).** *p < 0.05 / SAP: Systolic arterial blood pressure / DAP: Diastolic Arterial blood pressure / HDL-c: High density lipoprotein / TG: Triglycerides / LDL-c: Low density lipoprotein / TC: Total cholesterol / SAD: Sagittal abdominal diameter / WC: Waist circumference / BMI: Body mass index.

It was observed a positive correlation between SAD and systolic arterial blood pressure (r = 0.20), glycemia (r = 0.20), triglycerides (r = 0.32), LDL (r = 0.26), total cholesterol (TC) (r = 0.33), and a negative correlation with HDL-c (r = −0.21) (p < 0.05). It was observed a positive correlation between WC and systolic arterial blood pressure (r = 0.14), triglycerides (r = 0.31), total cholesterol (r = 0.21), and a negative correlation with HDL-c (r = −0.24) (p < 0.05). BMI showed a positive correlation with systolic arterial blood pressure (r = 0.22), total cholesterol (r = 0.20) and triglycerides (r = 0.23) (p < 0.05) (Figure 1).

## Discussion

The results of the present study showed that SAD correlated with more cardiovascular risk parameters than WC and BMI. WC and BMI correlated with four and three cardiovascular parameters, respectively, whereas SAD correlated with six out of seven parameters that were assessed in this study. Just like the present study, other researchers showed that SAD might be considered a good marker for metabolic blood disorders [16, 35].

In our study we observed that SAD is a better predictor of cardiovascular risk and metabolic syndrome components, if compared to BMI and WC. Other studies presented the very same correlation regarding SAD. It was observed that SAD was a better predictor for MS [36], but this is not unanimous, since that others studies showed that SAD, WC, and BMI had similar correlation with the parameters analyzed [20, 25].

WC has been the most commonly used abdominal adiposity marker due to the fact that it is easy to perform, has a very low cost and does not expose individuals to risks. The present study showed that WC correlated with SAP, HDL-c, TC, TG, and it was more efficient than BMI but not as much as SAD. Lopes de La Terra et al., observed that increased WC was strongly correlated with diabetes, but in the present study it was only observed for SAD [37].

it was showed in a cross-sectional study of over one hundred adults that only SAD was correlated with glycemia, TG, and HDL-c whereas TC and LDL-c did not correlate with any of the measurements [35]. In our study SAD showed correlation with PAS, glycemia, TC, TG, HDL-c and LDL-c. It was also more efficient than WC and BMI, thus proving that it is an adiposity marker that is more strongly correlated with cardiovascular risk factors. Other studies showed that SAD was the only anthropometric measurement that could predict insulin resistance, hyperinsulinemia, and glycemia [16, 38], thus making this measurement a strong insulin resistance marker, which was confirmed in our study.

We have assessed both WC and SAD to check which one would be more accurate to estimate visceral fat tissue (VFT), since it is correlated with various factors that were analyzed in the present study. VFT is the most active and it is also an insulin-resistant tissue. It also releases higher concentrations of adipokines that are related to pro-inflammatory processes, and contributes to the development of hypertension, insulin resistance, metabolic syndrome, and cardiovascular diseases [39–42]. The subcutaneous fat tissue (SFT) presents properties of intermediate order if compared to visceral fat, thus showing lower adipokine secretion [43].

WC generally evaluates the abdominal extension and it does not single out the two types of adipose tissue. SAD can perform a more accurate evaluation of VFT because its measurement is taken with the subject at supine position and the SFT tends to slide to the sides of the body due to its higher malleability. VFT is a more rigid tissue so it does not slide and makes the SAD a better analysis of VFT.

BMI is the most commonly used marker to assess nutritional status. In our study it only correlated with SAP, TC, and TG. Hence, BMI was not a strong marker to demonstrate the influence of adiposity upon the risk factor of cardiovascular diseases. It was already demonstrated that BMI presented weaker correlation with VFT if compared to SAD and WC [26, 44]. Two other studies showed that BMI was not as efficient as SAD and WC to identify insulin resistance in men [16, 45], which is in line with our study.

## Conclusion

We can conclude that SAD correlated with almost all the cardiovascular risk factors analyzed at present study. Thus, it is better to predict the quantity of abdominal fat tissue and cardiovascular risk; and should be employed in clinical practice.

## Authors’ original submitted files for images

Below are the links to the authors’ original submitted files for images. Authors’ original file for figure 1
