# Supplementary figures and images for: Sagittal abdominal diameter shows better correlation with cardiovascular risk factors than waist circumference and BMI
Source: J Diabetes Metab Disord. 2013 Jul 15;12:41. doi: 10.1186/2251-6581-12-41 (PMC3733622; doi:10.1186/2251-6581-12-41)

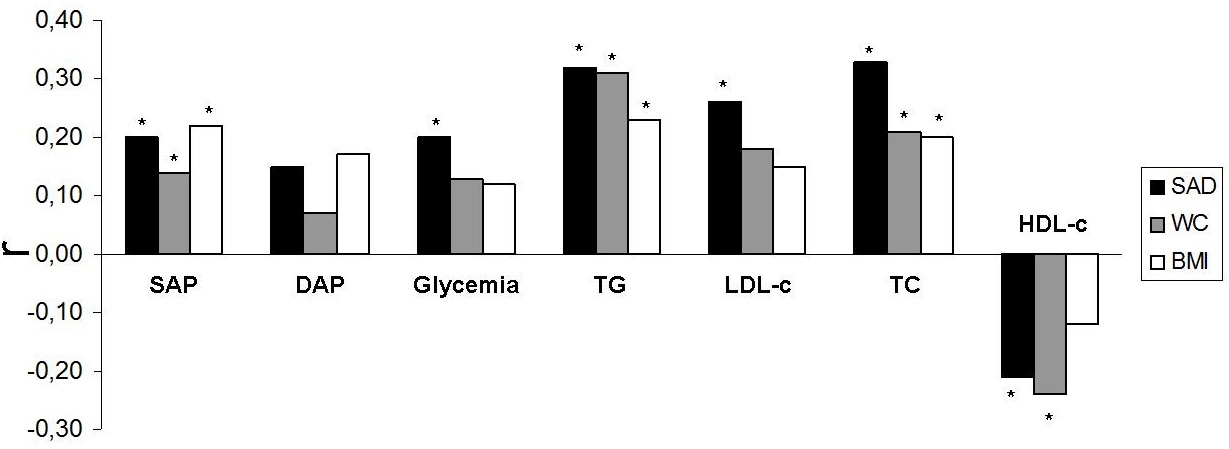

Supplement: Supplementary file 1 — Authors’ original file for figure 1 [file 40200_2013_67_MOESM1_ESM.jpeg]
